# Clinical Characteristics of Six Cases of Tracheobronchopathia Osteochondroplastica

**DOI:** 10.1155/2020/8685126

**Published:** 2020-06-16

**Authors:** Rong Guo, Mingming Zhou, Xiaohui Wei, Ling Niu

**Affiliations:** People's Hospital of Xinjiang Uygur Autonomous Region, Urumqi, Xinjiang, China

## Abstract

**Objective:**

To investigate the clinical characteristics of tracheobronchopathia osteochondroplastica (TO).

**Methods:**

The clinical data of six patients with TO from November 2016 to November 2018 were retrospectively analyzed. The etiology, clinical manifestations, diagnosis, and treatment of TO were summarized.

**Result:**

All six patients with TO were middle-aged males, confirmed by histopathological examination. The main clinical symptoms were cough, sputum, hemoptysis, chest pain, and repeated pulmonary infection. Some patients could make a preliminary diagnosis by chest CT, and bronchoscopy showed that TO mainly occurred in the trachea and the main bronchus and was more likely to invade the right bronchus. The treatment mainly includes anti-infection, phlegm-resolving, and other symptomatic treatment.

**Conclusion:**

TO is a benign disease predisposing to adults, and males are more likely to be affected. Its clinical manifestations are lack of specificity, and the cause may be related to chronic infection. Bronchoscopy combined with histopathological examination is the primary approach for the diagnosis of TO. There is no well-recognized treatment standard for TO, and the judgment of therapeutic effect is inconsistent. It is necessary to improve the understanding of this disease from a clinical perspective.

## 1. Introduction

Tracheobronchopathia osteochondroplastica (TO) is a rare benign disease. It refers to multiple nodular hyperplasia of bone or cartilage under the trachea and bronchial mucosa protruding into the lumen. The disease progresses slowly and can harden, narrow, or even block the trachea and bronchi [1]. There are about 600 cases of TO reported worldwide, in which 144 cases were reported from China, and the incidence of TO is around 0.5% until 2018 [2–4].

It is easy to misdiagnosis or unnotice TO because of the following reasons: the etiology and pathogenesis is not clear [5, 6]; the clinical symptoms are not typical; there are no specific characteristics for the high-risk population; and the clinicians have little knowledge about it. The first case of TO was recorded abroad in 1855. It was reported that there was no significant difference in the prevalence rate between men and women [7], while others reported that the prevalence rate was about 3 : 1 for men and women [8]. The pathogenesis of TO is unknown, which may be related to congenital tracheobronchial dysplasia, long-term physical, chemical, or mechanical stimulation, chronic inflammation, and metabolic disorders [6]. The most common clinical symptoms of TO include chronic cough and wheezing, and other nonspecific symptoms include chest tightness, shortness of breath, dyspnea, and hoarseness [9]. Patients with TO may be accompanied with lung cancer, tuberculosis, allergic bronchopulmonary aspergillosis, and other diseases [5].

The characteristic manifestations and pathological results under bronchoscopy are the “gold standard” for clinical diagnosis and determination of TO [10]. This paper reviews six cases of TO diagnosed by this standard in our hospital from November 2016 to November 2018 and analyses their clinical data. We found that the middle-aged patients were more likely to be affected, and males were more susceptible to this disease. Chronic infection such as tuberculosis and chronic sinusitis may be involved in the pathogenesis of TO; *Helicobacter pylori* infection may be related to the pathogenesis of TO, and further study is needed; bronchoscopy showed that TO mainly occurred in the trachea and the main bronchus and was more likely to invade the right bronchus. The abovementioned characteristics contributes to the study of TO, which may help clinicians improve their understanding of TO.

## 2. Methods

### 2.1. Clinical Data

From November 2016 to November 2018, a total of six patients with TO were admitted to the respiratory department of People's Hospital of Xinjiang Uygur Autonomous Region (PHXUAR). The data collected include age, gender, smoking history, occupation, time of onset, comorbidities, clinical symptoms, physical signs, routine blood test results, lung CT, lung function, and bronchoscopy.

### 2.2. Statistical Methods

Continuous and classified variables were analyzed by median and percentage, respectively.

## 3. Results

### 3.1. General Condition

All six patients were male and aged 41–54; three of them were civil servants, one was a company employee, and one was a freelancer. All patients had no long-term exposure to fumes, dust, irritant gases, and other special work history. Two patients had a long smoking history. One patient had a history of tuberculosis. One patient had a history of chronic sinusitis and nasal polyps. Two patients had a history of chronic atrophic gastritis, and their *Helicobacter pylori* infection was positive. One patient had a history of chronic cholecystitis for 20 years. One patient had a history of AF for 2 years(Table 1).

### 3.2. Clinical Characteristics

All six patients had symptoms of infection at the time of admission, but the duration of disease was different, and all patients were examined for sputum culture after admission. The results of sputum culture were negative except the second patient, which showed that the sputum culture for *Stenotrophomonas maltophilia* was positive, and this bacteria was sensitive to ceftazidime, levofloxacin, minocycline, and other antibiotics. The main clinical features of six patients are shown in Table 2.

### 3.3. Treatment

All six patients were given anti-infection, relieving cough and eliminating phlegm, hemoptysis, and other symptomatic treatment according to the clinical symptoms at the time of admission. Then, the symptoms were improved without special treatment such as surgery. All patients were followed up regularly in our outpatient department afterwards.

## 4. Discussion

Ossifying tracheobronchial disease is a rare benign lesion occurring in trachea and bronchus. Its clinical manifestations are not specific. In the past, ossifying tracheobronchial disease was often missed and misdiagnosed by clinician. With the development of electronic bronchoscopy, reports on ossifying tracheobronchial disease are increasing at home and abroad. Previous studies reported that the duration of TO was long, the onset age of TO was normally more than 50 years old, and there were occasional cases among adolescents and children [5]. Reports from overseas showed that there was no significant difference in the prevalence of TO between males and females [7]. It was also reported that the prevalence of TO between males and females was about 3 : 1 [8]. In China, there was no significant difference regarding the prevalence of TO between males and females in most studies [2]. In this report, six patients were male, all aged over 40 years old, which indicated that men are more susceptible to this disease.

The pathogenesis of TO is still unclear. There are several possible reasons, including congenital tracheobronchial dysplasia, long-term physical, chemical, or mechanical stimulation (fumes and irritating gases), chronic inflammation of tracheobronchial mucosa [6], metabolic disorders (endocrine hormone level or abnormal calcium and phosphorus metabolism), amyloidosis [11] and degeneration, and selective IgA deficiency [12]. The possible cause may also be the result of interaction between several reasons. In this report, two of five patients had a history of smoking. Studies in the past showed women had no history of smoking, but had a history of exposure to soot and dust. Thus, a long-term chronic inflammatory stimulation was also associated with TO. Long-term exposure to soot could stimulate airway mucosa, which may be one risk factor for the formation of TO. Jinyun et al. [13] reported that patients with TO had chronic rhinitis and chronic sinusitis at the same time, while Shipeng et al. [14] found that patients with TO accompanied with tuberculosis. Our study also showed that one patient had tuberculosis history in the past, and one patient had chronic sinusitis and nasal polyposis history, suggesting that chronic infection may be related to the pathogenesis of TO. In the past, some researchers have proposed that the formation of new bone and cartilage may be related to the transforming growth factor beta 1, a synergistic transformation of bone morphogenetic protein 2, endocrine hormone level, or abnormal calcium and phosphorus metabolism in vivo [15].

It is generally believed that TO develops slowly and has no specific clinical manifestations. The severity of the disease is related to the extent of the lesion and the degree of lumen obstruction. Common symptoms include cough, expectoration, chest congestion, and shortness of breath; rare symptoms include breathlessness, laboured breathing, hoarseness, foreign body sensation in the pharynx, and dysphagia [9]. Six patients in this report had no special clinical symptoms. It is reported [6] that a patient has many years of asthma history. He was misdiagnosed as bronchial asthma in adolescence and was diagnosed as TO by bronchoscopy in adulthood. In addition to pulmonary tuberculosis, chronic rhinitis, and chronic sinusitis, TO could also be accompanied by lung cancer, allergic bronchopulmonary aspergillosis, carbon sequestration, chronic atrophic gastritis, and other diseases. In this report, two patients with TO were accompanied with chronic atrophic gastritis and *Helicobacter pylori* infection history. Jinyun et al. [13] reported that one patient with TO was complicated with *Helicobacter pylori* infection. Therefore, whether *Helicobacter pylori* infection is related to the morbidity of TO needs further study. Yun et al. [16] reported that a patient's alveolar lavage fluid showed mild nonspecific cell clusters, which were easily confused with malignant tumors, but there was no follow-up results in the study.

There was no specific manifestation of pulmonary function in patients with TO. Some patients had normal pulmonary function, and some can also show restrictive or obstructive pulmonary ventilation dysfunction [12]. Of the six cases reported in this paper, two cases had normal pulmonary function as their pulmonary function was not affected due to the early stage of the lesion. Three patients indicated obstructive pulmonary ventilation dysfunction, and one reason maybe the proliferation of intrabronchial nodules, which results in narrow lumen and airflow limitation; another reason might be the combination of chronic obstructive pulmonary disease. One patient indicated restrictive pulmonary ventilation dysfunction, which may be caused by severe lesions and long duration, resulting in decreased compliance of lung tissue. This is similar to the results of lung function reported in previous studies [9]. Because the lesions of patients with TO are mostly located in the trachea and bronchus and do not involve lung parenchyma and alveolar wall tissue, the diffusion function of patients is usually normal.

Chest X-ray examination is not sensitive to the diagnosis of TO. CT examination is meaningful for the diagnosis of TO. CT can show multiple small nodules with or without calcification, which protrude from the trachea and main bronchial submucosa to the lumen. Nodules vary in size, and most lesions are located in the anterior and lateral walls of the trachea, which narrowed the lumen. When the lesion is serious, it can cause lumen collapse and atelectasis. It was reported that the tracheal membranes may also be involved in some patients with severe fusion, but it is also believed that the disease can be excluded if the membranous part is involved [3]. All six patients had right bronchial lesions with different manifestations, but it may suggest that TO is more likely to invade the right bronchi. However, there is no previous report on this issue, so more cases are needed for further confirmation. Thoracic MRI T1- and T2-weighted images can also be beneficial to the diagnosis of TO [17]. Diffuse trachea and diffuse irregular thickening of the main bronchial tube wall could be represented by moderate intensity signal; punctate calcification could be represented by low intensity signal; and coronal position could show the extent of wall thickening.

The manifestations of TO under bronchoscope are characteristic, which can determine the diagnosis and lesion range. Biopsy under bronchoscope is helpful for further diagnosis. Therefore, bronchoscopy is considered as the “gold standard” for clinical diagnosis of TO [10]. In recent years, the number of TO cases has increased gradually, which is closely related to the development and popularization of bronchoscopy technology. The typical manifestations are small nodules of different sizes and uneven distribution under the bronchoscope, which protrude from the trachea and bronchial submucosa to the lumen. The surface of the nodules is often covered with yellow or white secretions, which can be disseminated and fused into pieces when the lesions are serious. This may result in narrowing or beaded changes of the lumen. Typical lesions have a pebble-like appearance and nodular nature, which is difficult to get using biopsy forceps. Most of the lesions are located in the anterior and lateral walls of trachea and bronchus, and the lesions less likely invade glottis, supraglottic tissues, and tracheal membranes (posterior wall of the trachea). When the nodules are concentrated together, they can fuse into larger nodules, making the lumen narrow or even obstructed [18]. Some patients may have multiple bronchial polyps [19], which makes the stenosis of the lumen more obvious. The texture of TO lesion is hard. According to the literature, only 55% of patients can be diagnosed by the first histopathological biopsy, and crocodile forceps can be used for bronchoscopic biopsy to improve the positive rate of biopsy [12]. Typical pathological manifestations of TO were bone tissue or focal ossification under tracheobronchial mucosa, which served as the evidence for diagnosis of TO. Inflammatory cells such as lymphocytes and neutrophils infiltrated under mucosa, squamous epithelial metaplasia, and nonspecific hyperplasia could be also seen in some mucosa. Pathological findings in some patients with TO suggested keratinization of tracheal mucosal epithelium, which is the manifestation of different stages of the same disease, or suggesting the combination of other diseases, are not yet confirmed. Ihara et al. [20] found that fluorescent bronchoscopy can show squamous metaplasia of tracheal mucosal epithelium, which is helpful to improve the diagnostic rate.

There is no special treatment for TO at present. Patients with TO are prone to recurrent pulmonary infection and/or atelectasis. Treatment principles are anti-infection, drainage of airway secretions, inhalation of bronchodilators, inhalation of glucocorticoid spasmolysis, and anti-inflammation, which can alleviate the symptoms of patients [21, 22]. For some advanced lesions, the endoscopic interventional therapy or surgery can be used to relieve clinical symptoms [2]. Tracheal intubation is more difficult in patients with progressive TO [23].

TO is a benign disease, and among which male adults, particularly the middle-aged males, are more likely to suffer from it. There is a lack of specificity in clinical manifestations so far, and the right bronchus is more likely to be invaded. The causes may be related to chronic infection, including *Helicobacter pylori* infection. Chest CT can be used for the preliminary diagnosis of TO, and bronchoscopy and histopathological examination can be used for the final diagnosis. There is no well-recognized treatment guideline for TO worldwide at present, and symptomatic treatment is mostly adopted. However, there is still a lack of large-scale cases and multicenter, prospective studies to provide the evidence for the standardized diagnosis and treatment of TO.

## Figures and Tables

**Figure 1:**
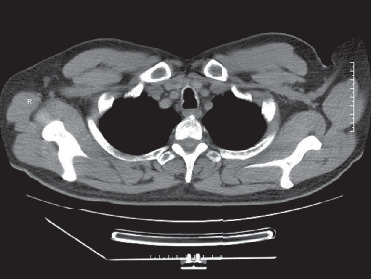
Case 3 lung CT: multiple ring calcifications in main bronchi walls.

**Figure 2:**
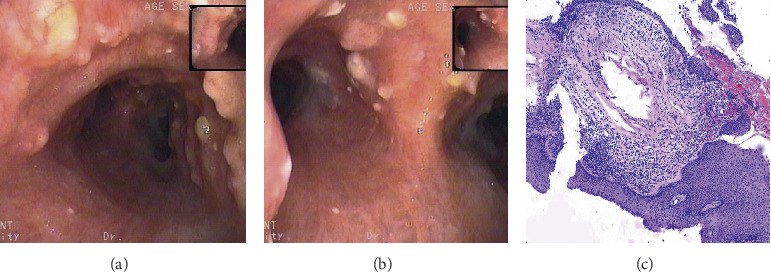
Case 4 (a) bronchoscopic examination: multiple nodular tuberositas of the lower trachea, the surface is uneven and pebble-like, part fuse into pieces. (b) Bronchoscopic examination: multiple nodular tuberositas of the tracheal carina and left and right main bronchus and some surfaces are covered with yellow pus. (c) Hematoxylin and eosin (H&E) stain at 100x magnification: pathological results of tracheal nodules, partial squamous cells on the bronchial mucosa epithelium, a large number of lymphocytes, plasma cells, and partial ossification.

**Table 1:** Summary of general condition.

General condition of patients	Statistical results (median/percentage)
Age	52.5 (41, 54)
Gender	6 (100%)
Smoking history	2 (33.3%)
Exposure to special surroundings	0
Course of disease (month)	7 (2.75, 111)
Comorbidities	—
Tuberculosis	1 (16.7%)
Chronic sinusitis and nasal polyps	1 (16.7%)
Chronic atrophic gastritis, *Helicobacter pylori* infection	2 (33.3%)
Chronic cholecystitis	1 (16.7%)
AF	1 (16.7%)

**Table 2:** Clinical features of six patients with TO.

No.	Age	Gender	Clinical manifestations	Physical signs	Pulmonary function tests	Lung CT	Bronchoscope examination results	Pathological results
1	54	Male	Cough, expectoration, and fever	Negative	Mild obstruction	The walls of trachea and bronchus were thickened, and the right main bronchus was collapsed and narrow	Nodular tuberositas of trachea and right main bronchus and crevice stenosis of the right main bronchus	Chronic inflammation of mucous membrane (right main bronchus) with visible bone tissue
2	54	Male	Cough and expectoration	Negative	Within normal limits	Bronchiectasis in the middle lobe of the right lung and multiple nodular shadows in the right lung	Multiple nodular tuberositas of trachea, carina, and left and right main bronchi	Chronic inflammation of mucous membrane (right main bronchus) with partial ossification
3	41	Male	Cough, expectoration, chest tightness, and shortness of breath	Negative	Within normal limits	Bronchiectasis in the middle lobe of the right lung, thickening of the trachea wall, and multiple circular calcification of the main bronchial tube wall (Figure 1)	Nodular tuberositas of left and right main bronchi, left lingual lobe, right upper lobe, and right middle lobe bronchi	Bronchial mucosa (right main bronchus) was infiltrated by acute and chronic inflammatory cells, and bone tissue with calcification can be seen partially
4	52	Male	Cough and expectoration hemoptysis	Negative	Mild obstruction	Flocculent shadow of the right middle lobe and left lingual lobe	Nodular tuberositas of the trachea, left and right main bronchus, and right middle bronchus (Figures 2(a) and 2(b))	(Left upper and lower lobe cristae) partial squamous cells on the bronchial mucosa epithelium, a large number of lymphocytes, plasma cells, and partial ossification (Figure 2(c))
5	41	Male	Cough and expectoration hemoptysis	Negative	Mild restriction	Bronchiectasis of both lungs with changes like the tree bud	Nodular tuberositas of the trachea, left and right main bronchi	Chronic inflammation of mucous membrane (right main bronchus) with mesenchyma and ossification
6	53	Male	Cough, expectoration, chest tightness, chest pain	Negative	Mild obstruction	Thickening of the trachea and right main bronchus wall and multiple ring calcification shadow	Nodular tuberositas of the trachea and right main bronchi	Chronic inflammation of mucous membrane (right main bronchus) with partial ossification

## Data Availability

The data used to support the study are available within the article.

## Additional Points

Established facts: Already known fact 1, tracheobronchopathia osteochondroplastica is a rare benign disease. Already known fact 2, tracheobronchopathia osteochondroplastica is characterized by unknown etiology, atypical, or asymptomatic clinical symptoms, so it could be easily misdiagnosed or missed diagnosis. Novel insights: New information 1, age fluctuation of 6 patients ranged from 41 to 54 years old, and most of them were middle-aged. New information 2, chronic infection may be involved in the pathogenesis of tracheobronchopathia osteochondroplastica diseases, such as tuberculosis and chronic sinusitis.
